# Affective Behavior in Parent Couples Undergoing Couple Therapy: Contrasting Case Studies

**DOI:** 10.3389/fpsyg.2021.634276

**Published:** 2021-03-18

**Authors:** Esther Liekmeier, Joëlle Darwiche, Lara Pinna, Anne-Sylvie Repond, Jean-Philippe Antonietti

**Affiliations:** ^1^Family and Development Research Center (FADO), Institute of Psychology, University of Lausanne, Lausanne, Switzerland; ^2^PROFA Foundation, Couples and Sexology Counseling Service, Lausanne, Switzerland

**Keywords:** couple therapy, couple interactions, affective behavior, coparental satisfaction, romantic relationship, observational coding

## Abstract

Being in a romantic relationship is characterized by a high degree of intimacy and affective involvement. Affective behavior indicates the emotional content in couple interactions and therefore promotes an understanding of the evolution of romantic relationships. When couples are also parents, their affective behavior reflects their romantic and coparental bonds. In this paper, we present an observation of parent couples’ affective behavior during a coparenting conflict discussion task to document whether and how much it improved during couple therapy. Two contrasting cases of affective behavior change are included. Observational coding of affective behavior within pre- and post-intervention coparenting conflict discussion tasks was carried out to compute means and CIs for each partner in both cases. In addition, the partners’ coparental and romantic satisfaction were evaluated through validated self-report questionnaires in pre- and post-intervention assessments; this helped document whether the partners’ coparental and romantic satisfaction were dissimilar between the two cases. Finally, a clinical analysis of both cases was realized with the contribution of the therapists to investigate possible differences within therapy sessions. Statistical analyses revealed negative means of affective behavior for couple A in the pre-intervention assessment and positive means in the post-intervention assessment. Partners from couple B had negative means of affective behavior in the pre- and post-intervention assessments. Results concerning coparental and romantic satisfaction differed: Couple A’s coparental satisfaction slightly increased and the romantic satisfaction somewhat decreased, whereas couple B’s coparental satisfaction remained stable and the romantic satisfaction slightly increased between the pre- and post-intervention assessments. The clinical analysis revealed that the interactional quality of couple A slightly improved within therapy sessions and that both partners succeeded in working together as coparents, notwithstanding their romantic distress. Couple B conveyed coparental distress and exhibited poor interactional quality throughout therapy sessions (e.g., repeated criticism and contempt). This study contributes to enriching the more traditional empirical research methods in the field of couple psychotherapy, as it takes into account microlevel affective changes within parent couples’ interactions in addition to self-reported data. Furthermore, the analysis of therapy sessions supports the importance of working with affective behavior in couple therapy.

## Introduction

Adult romantic partners experience intense emotions related to their relationships and have to cope with their emotional lives, both individually and as couples (Mirgain and Cordova, 2007; Sanford and Grace, 2011). When showing emotion, one communicates to their partner how they perceive a situation or might react (Sanford and Grace, 2011). Coan and Gottman (2007) defined the apparent and observable features of emotional content in couple interactions as affective behavior. Affective behavior can manifest itself in positive and negative nonverbal and/or verbal behaviors, such as affection, validation, interest, withdrawal, belligerence, and criticism (Coan and Gottman, 2007). Empirical literature shows that affective behavior is an important sign of what is going on in couple interactions (e.g., Gottman and Krokoff, 1989; Ben-Naim et al., 2013; Bloch et al., 2014). Previous research has demonstrated that couple interactions (specifically affective behavior) are linked with romantic satisfaction (e.g., Kim et al., 2007; Bloch et al., 2014).

In the context of parent couples, interactions between partners can reflect emotions experienced both in their romantic and coparental relationships. Romantic partners who are also parents share a romantic bond, but as they are responsible for the upbringing of one or more children, they are also bound by a coparental relationship (Feinberg, 2003). Existing data highlight that coparental interactions are linked with the coparental relationship. Prior research results have shown that positive coparental interactions (i.e., coparents being empathic and loving) are linked with a positive coparental relationship (i.e., coparental cooperation; Kolak and Volling, 2007).

Coparental interactions (i.e., interactions between two coparents regarding coparenting issues) have mainly been investigated within community samples, even though a significant number of couples seeking couple therapy are also parents (Klann et al., 2011). Therefore, studying improvements in coparental interactions and in the coparental relationship of parent couples undergoing couple therapy appears to be relevant. This study is an effort to investigate affective behavior in parent couple interactions in couple therapy and its relationship with the coparental and romantic relationships in a clinic setting.

In terms of associations between couple interactions in general (with both partners considered romantic partners or coparents), several outcomes can be found in couple research literature. The most widespread studies focus on the link between couple interactions and romantic satisfaction, showing that positive couple interactions are related to higher relationship satisfaction (e.g., Gottman and Krokoff, 1989; Rogge et al., 2006; Friend et al., 2017). Other studies have explored the link between couple interactions and outcomes, such as (1) depressive symptoms, in which negative interactions were related to higher reports of depressive symptoms (e.g., Brown and Harris, 2012); (2) family functioning, in which conflictual power dynamics in couple interactions were associated with lower family functioning (e.g., Lindahl et al., 2004); and (3) children’s reports of perceived threats and insecurity toward interparental conflict, in which negativity in parental conflict was linked with children’s perceptions of threats and insecure family representations (e.g., Zemp et al., 2016). Among this body of research, couple interactions have been investigated at various life stages, such as in the transition to marriage (e.g., Markman et al., 2010), transition to parenthood (e.g., Tanner Stapleton and Bradbury, 2012), or in elderly couples (e.g., Story et al., 2007). Furthermore, the majority of studies have been conducted within community samples, whereas others have addressed couple interactions within clinic samples.

Data specific to relationships between couple interactions using observational measurements and treatment responses within a clinic sample are indeed scarce. Previous research results concern the study of affective quality in general, without specifying the type of population (romantic or parent couples) or the addressed topic of discussion (romantic and/or coparental). One study of a sample of 55 married couples receiving behavioral or insight-oriented couple therapy showed that a lower proportion of nonverbal positive listening behaviors in a post-intervention conflict discussion task were associated with more distress 4 years after completing therapy (Snyder et al., 1993). Another study (Baucom et al., 2015) examined the link between couple interactions and treatment response as measured by relationship outcomes in a sample of 134 distressed couples randomly assigned to receive either integrative behavioral couple therapy or traditional behavioral couple therapy. Couples’ treatment responses were assessed based on their interactions during problem discussions (as rated by naïve coders) and the participants’ self-reports of romantic satisfaction. Results indicated (1) improvements in communication from pre- to post-therapy for couples in both therapeutic groups and (2) a positive link between improvement in couple communication and treatment outcomes. Thus, greater improvements in communication from pre- to post-therapy and better communication at post-therapy were related to better relationship outcomes. Given that a significant number of distressed couples initiating couple therapy are parents and that previous research conducted on clinic samples investigated affective quality in general without indicating whether the couples were in a romantic or coparenting relationship, further research is needed to explore coparental interactions of parent couples undergoing couple therapy.

Previous research has stressed the importance of considering the coparental relationship when studying romantic couples who also coparent. The act of coparenting involves coordination among adults responsible for the care and education of children (Feinberg, 2003). Coparental interactions have been studied in relation to several variables (e.g., child outcomes, family functioning, romantic satisfaction, and coparental satisfaction). One way to investigate coparental interactions of parent couples undergoing couple therapy is to explore the link between their affective behavior during a coparenting discussion and coparental satisfaction. Only a few studies have specifically evaluated this link in community samples. Findings relating to coparental affective interactions – either self-reported or observed – have shown an association between the quality of these interactions and of the coparental relationship. Kolak and Volling (2007, p. 468) investigated self-reported emotional expressiveness, which the authors define as reflecting “a stable pattern of how individuals communicate emotions within the family context”, and the quality of the self-reported coparental relationship in a sample of 57 community couples. Their results showed (1) positive links between fathers’ and mothers’ reported positive expressiveness (i.e., openness and being empathic, loving, and concerned) and perceived coparental cooperation as well as (2) positive associations between fathers’ and mothers’ reported negative expressiveness and perceived coparental conflict (Kolak and Volling, 2007). Hence, when partners reported experiencing more positive emotions and less negative emotions, they also appeared to perceive more cooperation and less conflict in their coparental relationship (Kolak and Volling, 2007). The second study consisted of an observation of parents’ affective interactions and the links between those interactions and observed coparenting behavior during family play. In a sample of 47 married community couples, McHale (1995) demonstrated an association between observed coparenting conflicts in couples’ interactions (i.e., partners blaming one another) in couple interviews, during which the parents were asked to discuss their home lives and the stresses experienced since the birth of their child/ren, and observed hostile-competitive coparenting within a family play situation. Results showed that partners blaming each other when interacting as a dyad were more likely to show hostile-competitive patterns of coparenting within the family, even after controlling for general romantic distress in the sample (McHale, 1995).

To date, studies on couples’ affective interactions have primarily been focused on interactions between romantic couples. However, in the context of parent couple interactions, both partners can be involved as romantic partners or coparents in discussing topics related to the upbringing of their child/ren. Furthermore, the partner’s affective behavior may be different in romantic or coparental interactions; for example, parent couples may be in conflict at the romantic level but share positive affective interactions at the coparental level or vice versa. To our knowledge, no data exist specifically concerning the quality of coparenting interactions in couple therapy settings. Therefore, further investigation within the field of clinical and couple psychology is needed to explore whether the results observed in community samples apply to particularly distressed couples, such as couples seeking help through couple therapy. To address these gaps in existing research, an ongoing randomized controlled trial (RCT) investigates the efficacy of an integrative brief systemic intervention for parent couples, specifically exploring coparental dynamics and their progress for parent couples undergoing couple therapy (de Roten et al., 2018). For the purpose of this study, two contrasting cases were drawn from the ongoing RCT sample of 65 parent couples based on the observation of the partners’ affective behavior within pre- and within post-intervention discussion tasks. The aims of this study were to: (1) explore observed affective behavior within pre- and post-intervention discussion tasks in which the parent couples discussed a disagreement regarding their coparental relationship to assess whether these couples could be differentiated on their affective behavior change, (2) analyze whether the different coparental affective behavior change patterns were also apparent in the pre- and post-intervention self-reported coparental and romantic satisfaction questionnaires, and (3) integrate the clinical analysis of the therapeutic processes of both cases to investigate whether the couple’s affective behavior change was also reflected in therapy sessions. Based on previous findings, we assumed that negative affective behavior would be associated with lower coparental and romantic satisfaction post-intervention. Moreover, we expected to identify explanatory markers of the couples’ change of positive and negative interactions within therapy sessions.

## Materials and Methods

### Participants

Both heterosexual couples were drawn from a sample of 65 parent couples participating in an ongoing RCT. Change patterns were calculated for a subsample of 25 couples based on available coded data for pre- and post-intervention affective behavior coding. Three change patterns were observed within the subsample: (1) nine couples experienced a positive change in their affective behavior; (2) eight couples did not undergo a change (i.e., their affective behavior remained positive or negative in both assessments); and (3) eight couples experienced a negative change. Couple A belonged to the group experiencing a positive change and couple B to the group with no change (their affective behavior remained negative in both assessments). Both couples were chosen from the subsample to: (1) compare affective behavior change in couples whose affective behavior was negative in the pre-intervention assessment, and (2) investigate whether a positive change vs. no change could also be observed in the couples’ questionnaires and therapy sessions. Data liable to identify the couples, such as name, age, profession, gender, and children’s ages, have been modified.

Partners from couple A, Marc and Emily, have been together for 8 years and have a 4-year-old son. They sought couple therapy because of issues related to their romantic intimacy. Couple B was composed of Arthur and Julia, who have been together for 35 years and have a 15-year-old daughter. Reasons for consulting were issues in their communication and disagreements regarding the upbringing of their child. Both couples were Swiss, living in Switzerland and belonging to the middle class. Each couple underwent a total of six systemic therapy sessions.

### Therapists and Treatment

Both therapists were experts in systemic therapy and clinical sexology. The couple therapy took place in a couple counseling service.

The therapists delivered brief systemic therapy to both couples. Brief systemic treatment refers to standard brief systemic couple therapy lasting from 6 to 12 months maximum. In our sample, each couple underwent a total of six therapy sessions, each approximately one month apart. This time interval provides enough time to initiate a process of change within the couple’s dynamic in between sessions and ensures that the therapist does not interfere negatively with the spontaneous change process (Selvini Palazzoli, 1980). This type of therapy mainly focuses on the romantic relationship and the difficulties couples face. However, therapists are likely to address other types of relationships, such as the parent-child or coparental relationship, as well as family functioning and families of origin. The therapists were free to use concepts and techniques from different schools of systemic psychotherapy, such as the structural, strategic, or transgenerational models (Haley, 1963; Minuchin, 1974; Selvini Palazzoli, 1988).

### Procedure

The study was conducted with the approval of the ethics committee of the University of Lausanne. Inclusion criteria for all participants from the ongoing RCT were that (1) partners were living together, (2) had at least one child not more than 16 years old, and (3) were involved in a coparenting relationship regarding the child or children. Couples were excluded from the study if they did not fulfill all three inclusion criteria or if they were in a crisis situation in which participation in the research could harm the therapeutic process. Participants were recruited through the clinics providing the treatment, and all gave written and informed consent to either audiotape or videotape the therapy sessions as well as to being filmed during couple discussion tasks before and after therapy. Before the first therapy session, a member of the research team contacted the couples to carry out the pre-intervention assessments. Participants filled out self-report questionnaires and took part in discussion tasks before the first therapy session and after the last. The pre- and post-intervention questionnaires were administered by the research team and completed privately by the participants. Therapy sessions took place in the clinic, while the discussion tasks took place either at the couples’ homes or in the clinic. In the observational discussion task (Gottman and Levenson, 1992; Baker et al., 2010), participants were asked to discuss a disagreement regarding their coparental relationship. Both parents received a list of topics related to coparenting (e.g., education, bedtime, outings, or mealtime). Each parent had to identify three disagreement topics, either from the list or they could write down their own. The research member conducting the task then collected the topic sheets and checked if the partners had a topic in common. If so, they suggested that the parents discussed the topic they had in common. If not, the research member selected a topic identified by one of the parents and asked the other parent if they would feel comfortable in discussing this topic. The couples received the following instruction: “Discuss [chosen subject], a topic on which you disagree as parents or that has caused arguments or tension. Start by discussing the subject and what could have caused the argument, and then try to think about ways to solve the disagreement. The objective is not that you end up finding one solution, but that you try to work together toward a resolution. You now have 5 min.” The procedure was repeated for the post-intervention discussion task. The couples were provided financial compensation for their participation at the end of the post-intervention measurements.

### Measures

#### Affective Behavior

Nonverbal and verbal affective behaviors within the pre- and post-intervention coparental discussion tasks were coded using an adapted version of the microanalytical Specific Affect Coding System (SPAFF; Gottman and Krokoff, 1989; Bodenmann, 2011). The SPAFF has been widely used and is an attested and externally validated approach to the coding of observational data, particularly for affective behavior in couples (Johnson, 2002; Zemp et al., 2017). This adapted system allowed the coding of discrete behaviors and is comprised of observational scales divided in five main categories: nonverbal positivity, nonverbal negativity, verbal positivity, verbal negativity, and neutral/nothing (Zemp et al., 2016). The verbal positivity category is composed of five subcategories: interest, validation, affect/caring, emotional disclosure, and constructive criticism. Verbal negativity consists of seven subcategories: criticism, defensiveness, domineering, stonewalling, speech interruption, contempt, and belligerence. The values for the various types of affective behavior in the positive subscale are hierarchical (interest = 1; constructive criticism = 5), with constructive criticism representing the person being the most emotionally involved in the conflict and thus a more negative affective behavior than interest/curiosity. The values for the various types of affective behavior in the negative subscale are also hierarchical (criticism = 6; belligerence = 12), with belligerence being the most intense negative affective behavior. The values of the nonverbal affective behavior categories are as well hierarchical (nonverbal positivity = 1; nonverbal negativity = 2). The value given for the category neutral/nothing was 88 and missing data were coded 99. The categories were coded separately for women and men, as previous literature has accounted for gender differences in communication patterns. The observational coding procedure involved three steps: (1) watching the video without coding, (2) coding the nonverbal behavior, and (3) coding the verbal behavior. These steps were repeated for the coding of the second partner. This coding method demonstrated good validity in previous studies (Kuster et al., 2015; Zemp et al., 2016, 2017), and rater teams achieved a high interrater reliability (i.e., Cohen’s kappa ≥ 0.90) in previous research (Zemp et al., 2017; Leuchtmann et al., 2019). A master coder from the University of Zurich trained the first author. After 12 h of training, 4 h of supervision, and 60 h of coding training tapes, the first author demonstrated high interrater reliability (i.e., Cohen’s kappa ≥ 0.90).

#### Coparental Satisfaction

The three dimensions of coparental satisfaction (support, conflict, and triangulation) were assessed with two questionnaires to get a comprehensive representation of this variable. The first questionnaire, the Parenting Alliance Measure (PAM), measured support, whereas the second, the Coparenting Inventory for Parents and Adolescents (CIPA), evaluated triangulation and conflict.

##### Parenting Alliance Measure

Coparental support was assessed by evaluating the strength of the perceived alliance between parents with the PAM (Konold and Abidin, 2001). The 20-item self-report questionnaire measured parenting aspects such as to what extent the parents are cooperative, communicative, and mutually respectful with regard to caring for their children. Scores on the PAM range from 20 to 100, with higher scores indicating a stronger and more positive parenting alliance. Internal consistency was excellent for mothers and fathers (mothers: *α* = 0.95; fathers: *α* = 0.95). We determined the Reliable Change Index (RCI) values for men and women using the data provided by Delvecchio et al. (2015): 15.11 for women and 15.29 for men.

##### Coparenting Inventory for Parents and Adolescents

The parents’ perceptions of conflict and triangulation were measured with the 16-item CIPA (Teubert and Pinquart, 2011). Scores range from 0 to 4, with higher scores indicating more conflict and triangulation. Internal consistency was good for mothers and fathers (mothers: *α* = 0.84; fathers: *α* = 0.87). Following recommendations of Jacobson and Truax (1991), we calculated the RCI values for men and women using the data provided by Teubert and Pinquart (2011): 2.06 for women and 1.78 for men.

#### Romantic Satisfaction

The quality of the romantic relationship was evaluated with the 32-item Dyadic Adjustment Scale (DAS; Spanier, 1976). The global adjustment scores range from 0 to 151, with higher scores indicating a better adjustment. Scores underneath the cut-off score of 97 (Jacobson and Truax, 1991) and indicate that the partner is experiencing distress in the romantic relationship. Internal consistency was excellent for women and good for men (women: *α* = 0.91; men: *α* = 0.89). Following recommendations of Jacobson and Truax (1991), we calculated the RCI values for men and women using the data provided by Baillargeon et al. (1986): 12.2 for women and 13.51 for men.

### Statistical Analyses

The observational data were entered in R (R Core Team, 2020), and the categories of the nonverbal behavior were re-coded as follows: positive nonverbal behavior = 1, negative nonverbal behavior = −1, and neutral/missing behavior = 0. Verbal behavior was re-coded as follows: Negative verbal affective behaviors were characterized by negative numbers (criticism = −1, defensiveness = −2, domineering = −3, stonewalling = −4, speech interruption = −5, contempt = −6, and belligerence = −7), thus representing gradually more negative affective behaviors. Positive numbers were used to identify the positive verbal affective behaviors (constructive criticism = 1, emotional disclosure = 2, affect/caring = 3, validation = 4, and interest/curiosity = 5), with higher numbers illustrating that the person displayed a more positive affective behavior. Each partner’s nonverbal and verbal behavior raw scores were separately plotted within the pre- and post-intervention assessments. For each time interval, the vertical unit matched the affective behavior code displayed by the participant, and the horizontal distance unit matched the time sequence. Therefore, if the affective behavior was positive, the point was above zero, and if the affective behavior was negative, the point was below zero. Greater numbers indicate more intense affective behavior. Means and CIs were computed for each partner, and the mean affective behavior of each partner was represented by a horizontal line in the plots. Then, paired student *t*-tests were calculated to contrast the partners’ means between the pre- and post-intervention assessments. The null hypothesis stipulated both means to be equal, while the alternative hypothesis postulated a difference between the means.

### Clinical Analysis

The clinical analysis was conducted in two steps, after the coding of the affective behavior. First, the first two authors (both psychotherapy researchers) summarized and analyzed all audiotaped therapy sessions (i.e., six sessions for each couple). Within each therapy session, particular attention was paid to specific markers, such as the couple’s affective interactional dynamics (e.g., voice tone, specific verbal cues, and speaking turns) and the therapist’s interventions (e.g., work on the romantic and/or coparental relationship, downregulation of the couple’s negative interaction cycles, and work on the couple’s affective behavior dynamics). Then, in the second step, these analyses were shared with the two therapists who validated the analyses or suggested revisions (e.g., they refined the content or gave additional information on the couple’s affective interactional dynamic).

## Results

Results are presented in three parts: affective behavior change, coparental and romantic satisfaction changes, and clinical analysis.

### Affective Behavior Change

**Figure 1:**
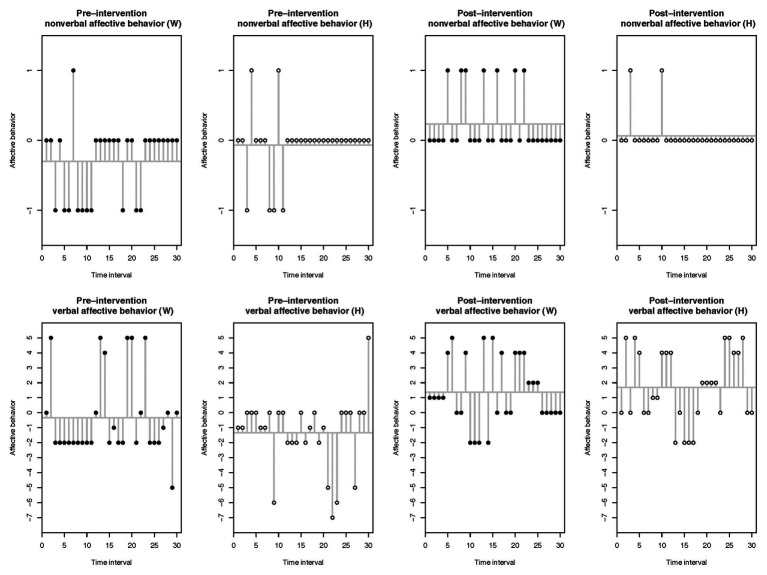
Couple A: raw scores of the observed nonverbal and verbal affective behavior within the pre- and post-intervention discussion tasks.

**Figure 2:**
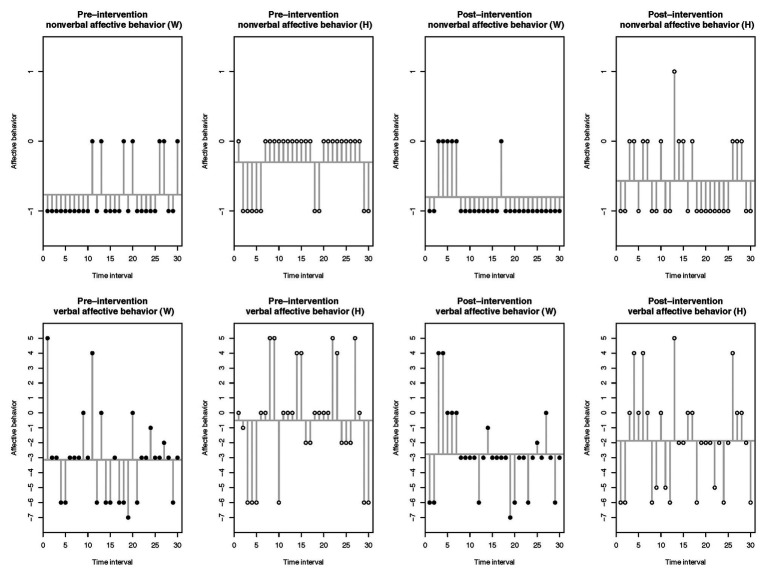
Couple B: raw scores of the observed nonverbal and verbal affective behavior within the pre- and post-intervention discussion tasks.

The plotted raw scores for couple A, as depicted in Figure 1, indicate that the partners’ nonverbal and verbal affective behavior was substantially negative within the pre-intervention discussion task and mainly positive within the post-intervention discussion task. Regarding couple B’s plotted raw scores, both partners’ nonverbal and verbal affective behavior were above all negative within the pre- and post-intervention discussion tasks, as illustrated by Figure 2. For both figures, the time interval is represented on the *X*-axis and the raw scores of affective behavior on the *Y*-axis.

Table 1 displays results of the partners’ affective behavior means and their respective CIs. The means summarize each partner’s nonverbal and verbal affective behavior in terms of the 5-min discussion task. Analyses showed negative means in the nonverbal and verbal affective behavior for partners from couple A within the pre-intervention discussion task and positive means for the nonverbal and verbal affective behavior of both partners within the post-intervention discussion task. Results for couple B indicated negative means for both partners in the nonverbal and verbal affective behavior within the pre- and post-intervention discussion tasks.

**Table 1:** Means and CIs for nonverbal and verbal affective behavior within the pre- and post-intervention discussion tasks for couples A and B.

	Couple A		Couple B	
Affective behavior	*M*	95% CI	*M*	95% CI
Pre-intervention
Woman				
Nonverbal	−0.30	(−0.50, −0.10)	−0.77	(−0.93, −0.61)
Verbal	−0.33	(−1.38, 0.72)	−3.13	(−4.21, −2.06)
Man				
Nonverbal	−0.07	(−0.23, 0.10)	−0.30	(−0.47, −0.13)
Verbal	−1.33	(−2.23, −0.43)	−0.50	(−1.85, 0.85)
Post-intervention
Woman				
Nonverbal	0.23	(0.07, 0.39)	−0.80	(−0.95, −0.65)
Verbal	1.37	(0.53, 2.21)	−2.77	(−3.77, −1.77)
Man				
Nonverbal	0.07	(−0.03, 0.16)	−0.57	(−0.78, −0.35)
Verbal	1.70	(0.80, 2.60)	−1.87	(−3.12, −0.61)

To compare each partner’s affective behavior change between the pre- and post-intervention discussion tasks, we computed paired student *t*-tests. For couple A, results revealed that the woman displayed substantially more positive nonverbal and verbal affective behavior in the post-intervention discussion task [nonverbal: *t*(29) = −3.76, *p* < 0.001, 95% CI (−0.82, −0.24); verbal: *t*(29) = −2.66, *p* = 0.013, 95% CI (−3.01, −0.39)]. Even though the man’s non-verbal affective behavior mean was positive in the post-intervention assessment, analyses indicated that his mean did not differ from the pre-intervention assessment [*t*(29) = −1.44, *p* = 0.161, 95% CI (−0.32, 0.06)]. However, the man showed significantly more positive verbal affective behavior in the post-intervention assessment [*t*(29) = −5.01, *p* < 0.001, 95% CI (–4.27, –1.79)]. For couple B, results from the paired student *t*-tests suggested that the woman’s nonverbal and verbal behavior means did not differ in the post-intervention discussion task [nonverbal: *t*(29) = 0.27, *p* = 0.79, 95% CI (−0.22, 0.28); verbal: *t*(29) = −0.48, *p* = 0.636, 95% CI (−1.93, 1.20)], thus suggesting that her nonverbal and verbal affective behavior remained negative in the post-intervention assessment. The man showed significantly more negative nonverbal affective behavior in the post-intervention discussion task [*t*(29) = 2.11, *p* = 0.043, 95% CI (0.01, 0.52)], whereas there was no difference in his mean verbal affective behavior [*t*(29) = 1.35, *p* = 0.188, 95% CI (−0.71, 3.44)], therefore indicating that his verbal affective behavior stayed negative.

### Coparental and Romantic Relationship Satisfaction Changes

Table 2 displays coparental and romantic satisfaction scores for couples A and B in the pre- and post-intervention assessments. In the post-intervention assessment, couple A reported a more positive coparenting alliance and less conflict and triangulation, as well as less romantic satisfaction. In couple B, the woman reported a similar and the man a lower score of coparenting alliance and both reported less conflict and triangulation. In addition, both partners reported higher scores of romantic satisfaction. Although partners from couples A and B reported changes in their coparental and romantic satisfaction, none of these can be considered as clinically significant.

**Table 2:** Pre- and post-intervention scores of coparental and romantic satisfaction for couples A and B.

	Couple A						Couple B					
	Woman			Man			Woman			Man		
	Pre	Post	Δ	Pre	Post	Δ	Pre	Post	Δ	Pre	Post	Δ
Alliance	72.0	78.0	6.0	85.0	96.0	11.0	76.0	76.0	0.0	85.0	82.0	−3.0
Conflict and triangulation	0.6	0.2	−0.4	0.8	0.3	−0.5	1.6	1.3	−0.3	1.7	1.1	−0.6
Romantic satisfaction	83.0	77.0	−6.0	95.0	90.0	−5.0	96.0	99.0	3.0	109.0	112.0	3.0

Δ corresponds to the score difference between the post-intervention and pre-intervention assessments. Alliance scores range from 20 to 100, with higher scores indicating a more positive alliance; Reliable Change Index (RCI) values were 15.11 for women and 15.29 for men (Delvecchio et al., 2015). Conflict and triangulation scores range from 0 to 4, with higher scores suggesting more conflict and triangulation; RCI values were 2.06 for women and 1.78 for men. Romantic satisfaction scores range from 0 to 151, with higher scores showing a better adjustment; RCI values were 12.2 for women and 13.51 for men.

### Clinical Analysis

Specific change markers within therapy sessions, such as the couples’ interactional dynamics, were identified to shed light on the couples’ affective behavior analysis. The results revealed that Marc and Emily (couple A) were able to foster a supportive coparenting relationship despite still experiencing romantic distress at the end of therapy. Their interactional dynamic underwent a slight positive change throughout the therapy sessions. For Arthur and Julia (couple B), the clinical analysis revealed the continuous presence of several coparental conflicts throughout the therapy sessions, which mainly remained irreconcilable. The interactional dynamic stayed negative, with the presence of frequent criticism and contempt throughout the therapy sessions.

#### Couple A

Marc and Emily’s therapy indicated that they shared a supportive coparental bond, even though their romantic distress remained. Faced with a couple who came to therapy highly romantically distressed, the therapist sought to support and strengthen their coparental resources to preserve the coparental relationship. More broadly, the therapist also worked on the couple’s interactional dynamic: e.g., Marc frequently criticized Emily, and Emily was mainly closed off and sometimes defensive. This interactional dynamic changed throughout therapy sessions, and at the end of the therapy, Marc was more validating and Emily became more assertive.

During the first session, the therapist was confronted with two different demands and a highly negative and destructive interactional dynamic. When the therapist explored both demands, it appeared that Marc wished for more physical intimacy and sex, whereas Emily desired less tension and more dialog in general. The nature of the couple’s conflict around their romantic life was related to sexual desire discrepancies. During the couple’s interactions within the first session, both partners generally expressed themselves in monologues (i.e., both spoke to the therapist and not to one another); additionally Marc often overtly criticized Emily in front of the therapist, while Emily often broke down in tears and did not speak.

In the following therapy sessions, the therapist worked on the partners’ demands and explored their needs. Unfortunately, it appeared that the deleterious interactional dynamic between the partners challenged the progression of the couple’s romantic relationship. For instance, in Session 3, Marc overtly criticized Emily’s general knowledge in front of the therapist. As a response to Marc’s aggressive behavior, Emily started crying and tried to defend herself, but she often could not finish her sentences. The therapist also explored the coparental relationship through the couple’s transition to parenthood and everyday life. It seemed that the atmosphere lightened when Marc and Emily tackled coparental topics within therapy sessions; both partners agreed more and sounded less tense. Given this context, the therapist put her focus on the positive aspects of the couple’s relationship – for instance, their coparental relationship – and worked on soliciting and reinforcing this resource.

In the last therapy session, the therapist and the couple investigated the couple’s progress during the therapy. It seemed that, notwithstanding the couple’s romantic distress and the impossibility of reconciling both partners’ demands, Marc and Emily’s interactions changed positively throughout therapy. Both partners recollected communicating substantially more throughout therapy sessions. Furthermore, Emily confirmed that the sessions helped her open up and become more assertive. As for Marc, he seemed to be able to listen more and to validate his partner’s feelings to a greater extent. The therapist supported and validated this improvement. Finally, both partners felt they had made a step toward improvement and did not feel the need to continue therapy. Therefore, the therapy stopped after six sessions.

#### Couple B

Julia and Arthur’s therapy analysis indicated they had several disagreements about their romantic and coparental relationships that could not be solved through therapy. It appeared that Julia and Arthur had different expectations of their romantic relationship and dissimilar educational values regarding their daughter’s upbringing. Confronted with the repeated presence of criticism and contempt within the couple’s interactions, the therapist attempted to reduce the negative interactional dynamic throughout therapy sessions. Moreover, the therapist sought to explore and reconcile both partners’ needs. Nonetheless, this conflict and negativity appeared to have been in place for a long time in the couple’s interactional dynamic and did not change in spite of the therapy sessions.

In the first session, the therapist’s exploration of both partners’ goals for therapy showed that they came because of their recurrent problematic communication and frequent disagreements in their everyday life. Further exploration indicated that Julia was the main source of the therapeutic demand: she wished for the couple’s problematic interactions to change. During this session, the therapist was confronted with Julia and Arthur’s conflicts and lack of empathy toward each other; therefore, she intervened to comment on the negative dynamic between the couple and worked on reducing their conflicts in both their romantic and coparental relationships.

In subsequent sessions, the couple’s interactional dynamic remained generally negative. Julia and Arthur appeared to communicate high coparental distress and exhibit poor interactional quality when interacting in therapy sessions. Both partners frequently criticized and interrupted each other and showed a substantial lack of empathy toward each other by exchanging dismissive remarks. The therapist worked on the couple’s goals (i.e., changing the negative interactional dynamic) by intervening and reframing the couple’s interactions. For instance, the therapist used the “positive connotation technique” (i.e., responding from another angle to a patient’s statement by re-labeling in a positive way a situation that was initially labeled negatively). This means intervening in the following way: the therapist interrupted an argument and meta-communicated about what was happening by saying that the ongoing conflict was a sign that their relationship was still important to both of them. This allows partners to view their conflict in a different way and is seen in the systemic approach as a lever for change (Haley, 1963; Jackson, 1968; Selvini Palazzoli et al., 1978). The therapist also explored the couple’s coparental functioning during the transition to parenthood and in their everyday life. It turned out that Julia and Arthur seemed not only to have different needs but also dissimilar or even opposed educational values. To reconcile both partners’ needs and values, the therapist explored each partner’s motivations to hang on to their individual values. In positively reframing the contributions of both partners by saying that they actually pull on the same string but not at the same time, the therapist worked on promoting a sense of unity between the coparents to strengthen the coparental relationship.

In the last session, Julia and Arthur argued anew about topics related to their coparental relationship, as was generally the case throughout therapy. This detrimental interactional dynamic led the therapist to interrupt both partners on several occasions to reduce the tension between them. At the end of the session, the therapist encouraged the couple to work together toward a solution by identifying what they could do to communicate their needs better and adapt to their partner’s needs. As no significant change had occurred within the couple’s interactional dynamic – and due to the couple’s willingness to continue working on their demands – the therapist and the couple agreed to schedule additional therapy sessions outside of the research frame.

## Discussion

Results from the contrasted cases indicate that the affective behavior change patterns that could be observed in the coparental discussion tasks (positive change vs. no change) were not systematically related to similar coparental and romantic questionnaire results. Couple A displayed a positive affective behavior change in the coparental discussion task which was reflected in the coparental satisfaction questionnaire but not in the romantic satisfaction questionnaire. Couple B’s affective behavior change remained negative after therapy in the coparental discussion task, whereas both partners reported moderately high coparental satisfaction both in the pre- and post-intervention questionnaires and their romantic satisfaction increased between the pre- and post-intervention assessments.

The association between couple A’s positive change of affective behavior and the increase in the coparental satisfaction questionnaires is in line with previous research demonstrating that more positive coparenting interactions are related to a higher quality of coparental relationship (Baker et al., 2010). It is of interest to note that the coparental positive change was stronger in the affective behavior microlevel coding than in the self-report questionnaires, which suggests that microlevel analysis gives results that are slightly different from self-reported measurements. The fact that couple A’s positive affective behavior change was not reflected in the romantic satisfaction questionnaires contrasts with previous research showing that the quality of couple interactions is associated with the quality of romantic satisfaction (e.g., Rogge et al., 2006). Hence, we can assume that changes in couple A’s affective behavior are not just as much a function on an improvement in overall satisfaction.

Couple B’s results contrast with previous research suggesting that negative interactions are related to hostile-competitive coparenting (McHale, 1995) and lower relationship satisfaction (Friend et al., 2017). A discrepancy is therefore also observed here between observational results and questionnaires. Self-report questionnaires provide information on an individual’s perceptions, whereas observational methods capture relational dynamics by providing direct data on them (Baucom and Crenshaw, 2019). Therefore, data collected *via* observational coding by a third party are also independent from potential memory or social desirability bias, which could be present in couple B’s self-reports. Finally, this gap in the results highlights that observational measurements enable researchers to capture unique and specific dynamics of couples’ interactions, which provide additional information to data collected through self-report measures. Therefore, future studies should consider more frequently integrating observational methods in addition to self-report measurements to investigate couple interactions (Darwiche and de Roten, 2015).

The clinical analysis showed that the interactional dynamic of couple A slightly and positively evolved within therapy sessions. Marc and Emily’s coparental interactions and relationship seem to have been reinforced during therapy. However, their romantic distress remained after terminating therapy. We could hypothesize that during therapy sessions, the couple recognized their coparental relationship as a strength which might have led them to consolidate their coparental interactions and relationship. Both parents may have been particularly motivated to improve their coparenting relationship for their children’s benefit. For couple B, the clinical analysis revealed the presence of several coparental conflicts that could not be settled during the six therapy sessions. We can hypothesize that the brief therapeutic setting might not have been enough psychoeducational and suitable for a couple that appeared chronically distressed.

Taken together with previous research, our study was intended to explore processes within the coparental relationship in addition to those present in the romantic relationship in a sample of parent couples undergoing couple therapy. To date, empirical literature describing how communication influences relationship outcomes has mainly focused on interactions taking place within romantic relationships and their links with romantic satisfaction. Investigating the evolution of the coparental relationship remains an atypical scope in couple therapy. Our findings support previous research results indicating that the coparental and romantic relationships do not necessarily evolve jointly (Le et al., 2016). Therefore, future studies should consider exploring the romantic and coparental relationships separately.

In the context of frequent separations between couples, research efforts highlighting changes in the coparental relationship within couple therapy appear highly relevant and important (for a systematic review and meta-analysis of coparenting programs, see Eira Nunes et al., 2020). Literature has demonstrated broadly that coparental satisfaction is significantly linked with well-being, child rearing, and child adjustment (Bodenmann, 2016). Parents having constructive coparental interactions and reporting satisfaction in their coparental relationship seem more likely to define parenting goals together and provide mutual support related to child rearing (Holland and McElwain, 2013). Finally, results from a meta-analysis underline that coordination among adults responsible for the care and education of children is significantly related to fewer internalizing and externalizing symptoms within their child/ren (Teubert and Pinquart, 2010). Therefore, reinforcing the coparenting relationship can constitute a protective factor for children whose parents consider separation or divorce.

The present study has some limitations. First, this contrasting case study is a first exploration and step, and the results will need to be replicated with a subsample of 65 parent couples for whom data for pre- and post-intervention analyses of affective behavior are available within the ongoing RCT’s expected total sample of *N* = 80 couples with pre-post intervention data. This will allow further testing of our hypothesis that affective behavior in parent couples’ interactions before entering couple therapy could be predictive of their progress in romantic satisfaction, coparental satisfaction, and overall individual symptomatology (e.g., propensity to anxiety and depressive symptoms) between pre- and post-intervention assessments. Second, due to the small sample size, we analyzed the partners’ nonverbal and verbal affective behavior independently. Nonetheless, as our data were drawn from couples, we can still postulate an interdependence and interconnectedness within our findings. The broader sample from the ongoing RCT will additionally make it possible to: (1) use data analytic models specifically suited to dyadic data, such as actor-partner interdependence models or growth-curve modeling (Kenny et al., 2006), and (2) analyze different patterns of affective behavior change, including a positive to negative affect behavior change. Third, we cannot rule out that a therapeutical approach focused on affective behavior (e.g., Halford et al., 2003; Gottman and Schwatz Gottman, 2008; Bodenmann et al., 2014) might have led to other results. The systemic approach incorporates the observation of affective behavior; nevertheless, it does not involve systematic therapeutic work on this aspect as do other models. One limitation is that the therapists had general guidelines for their interventions, which makes it difficult to know whether the treatment received by the couples was comparable. Another important limitation is related to the fact that only the first author coded the affective behavior; this limitation is balanced by the fact that the first author was qualified as an expert coder. Furthermore, the affective behavior coding might have influenced the clinical analysis, given that it was conducted by the same members of the research team. However, the potential bias is compensated by the therapists’ contribution to the clinical analyses. Finally, we cannot exclude the possibility that external factors or factors specific to the participants influenced the results, such as between-session events or participants’ disposition toward change, as we only integrated an analysis of the processes within therapy sessions.

Our study is a first step toward investigating coparental relationships through observed coparental interactions with parent couples within a clinical setting. Observing couples’ interactions makes it possible to apprehend a couple’s conflict in a somewhat realistic setting, compared to self-report measures. The results are therefore meaningful to clinicians and clinical training. Previous research has stressed the importance of teaching clinicians to detect negative nonverbal affective behavior within couples’ interactions (Patterson et al., 2012). Our results can prompt couple therapists on the importance of considering micro-observational research results on nonverbal and verbal affective behavior to allow them to identify their clients’ affective behavior changes. In the last few decades, research has highly been influenced by narrative therapy and other postmodern approaches, and their reluctance to observe, comment upon, and intervene with couple’s interactive behavior. Hence, our study can contribute to the existing body of research that focuses on specific practices for working with affective exchanges in couple therapy (e.g., Epstein and Zheng, 2017; Johnson, 2020).

## Data Availability Statement

The raw data supporting the conclusions of this article will be made available by the authors, without undue reservation, to any qualified researcher.

## Ethics Statement

The studies involving human participants were reviewed and approved by Ethics Committee of the University of Lausanne. The patients/participants provided their written informed consent to participate in this study.

## Author Contributions

JD contributed to the conception and design of the study. EL and J-PA performed the statistical analyses. EL and JD contributed equally to writing of the manuscript. J-PA, A-SR, and LP read and revised sections of the manuscript. All authors contributed to the article and approved the submitted version.

### Conflict of Interest

The authors declare that the research was conducted in the absence of any commercial or financial relationships that could be construed as a potential conflict of interest.
